# Redundancy of the genetic code enables translational pausing

**DOI:** 10.3389/fgene.2014.00140

**Published:** 2014-05-20

**Authors:** David J. D'Onofrio, David L. Abel

**Affiliations:** ^1^Control Systems Modeling and Simulation, General DynamicsSterling Heights, MI, USA; ^2^Department of Humanities and Science, Math Department, College of Humanities and Science, University of PhoenixDetroit, MI, USA; ^3^Department of ProtoBioCybernetics/ProtoBioSemiotics, The Gene Emergence Project of The Origin of Life Science Foundation, Inc.Greenbelt, MD, USA

**Keywords:** algorithm, translational pausing, ribosome, regulation, co-translational folding, Shine Dalgarno sequences, degeneracy, multi-dimensional code

## Abstract

The codon redundancy (“degeneracy”) found in protein-coding regions of mRNA also prescribes Translational Pausing (TP). When coupled with the appropriate interpreters, multiple meanings and functions are programmed into the same sequence of configurable switch-settings. This additional layer of Ontological Prescriptive Information (PI_o_) purposely slows or speeds up the translation-decoding process within the ribosome. Variable translation rates help prescribe functional folding of the nascent protein. Redundancy of the codon to amino acid mapping, therefore, is anything but superfluous or degenerate. Redundancy programming allows for simultaneous dual prescriptions of TP and amino acid assignments without cross-talk. This allows both functions to be coincident and realizable. We will demonstrate that the TP schema is a bona fide rule-based code, conforming to logical code-like properties. Second, we will demonstrate that this TP code is programmed into the supposedly degenerate redundancy of the codon table. We will show that algorithmic processes play a dominant role in the realization of this multi-dimensional code.

## Introduction

Genomic prescriptions of biofunctions are multi-dimensional. Within the genome domain, executable operations format, read, write, copy, and maintain digital Functional Information (FI) (Szostak, 2003; Carothers et al., 2004; Hazen et al., 2007; Schrum et al., 2010; Sharov, 2010). Bio-molecular machines are programmed to organize, regulate, and control metabolism.

The genetic code is composed of data sets residing in the particular sequencing of nucleotides (Abel and Trevors, 2005, 2006). Data sets are found in both the coding and non-coding regions of the DNA (Mercer et al., 2009; Craig and Wong, 2011; Ghanbarian et al., 2011; Derrien et al., 2012; Hunter et al., 2012; Morris, 2012; St. Laurent et al., 2012; Bucher, 2013; Arrowsmith et al., 2012). Here, we limit our discussion of data sets to those strings or sequences of codons prescribed in the coding regions of the DNA molecule. We will show that coding for proteins is not the only form of biological PI generated by these regions.

The central dogma of protein synthesis involves both transcription and translation processes to synthesize protein products. Protein folding has been studied for over 50 years. A large percentage of protein-folding is assisted by chaperones (Hartl and Hayer-Hartl, 2009; Giffard et al., 2013), some of which are RNAs rather than protein chaperones (Hyeon and Thirumalai, 2013). But the final fold is primarily constrained by the primary-structure of amino-acid sequence. Over the last 30 years, studies have shown that protein-coding sequencing significantly affects translation rate, folding and function (Pedersen, 1984; Andersson and Kurland, 1990).

Most protein functionality is dependent upon its three-dimensional conformation. These conformations are dependent upon folding mechanisms performed upon the nascent protein. Such folding mechanisms recently have been linked directly to several cooperative translational processes (Kramer et al., 2009; Li et al., 2012). By “translational processes,” we mean processes that go beyond simply translating and linking the amino acids. This paper expands the understanding of translation processes to go beyond just the mechanistic interactions between the polypeptide and ribosome tunnel. Internal mechanisms involving mRNA interactions occur by extension. Chaperone function occurs as an external mechanism. These mechanisms all work to contribute coherently to the folding process. The crucial point is that they are all dependent upon momentary pauses in the translation process. We collectively define these linked phenomena and their rate regulation as “co-translational pausing.” The dependency of folding on these multiple translation processes has been defined as “co-translational folding” (Netzer and Hartl, 1997; Hardesty et al., 1999; Nicola et al., 1999; Kolb et al., 2000; Sakahira and Nagata, 2002; Oresic et al., 2003; Johnson, 2005; Komar, 2009; Zhang et al., 2009; O'Brien et al., 2010, 2012; Saunders et al., 2011; Zhang and Ignatova, 2011; Krobath et al., 2013). The meaning of these concepts will be expanded later in this section. They reveal the ribosome, among other things, to be not only a machine, but an independent computer-mediated manufacturing system (Stahl et al., 2002; Liao and Seeman, 2004; Spirin, 2004; Rodnina et al., 2007; Church, 2009; Gao et al., 2009; Hu et al., 2009; Frank and Gonzalez, 2010; Johnson, 2010; McIntosh, 2010).

Nucleotide, and eventually amino acid, sequencing are both physicodynamically indeterminate (inert) (Rocha, 2001; Rocha and Hordijk, 2005). Cause-and-effect physical determinism, in other words, cannot account for the programming of sequence-dependent biofunction (Abel and Trevors, 2005, 2007; Abel, 2007, 2009a,b, 2010, 2011a, 2012b). Nucleotide sequencing and consequent amino acid sequencing are formally programmed in both the nascent protein, and in the chaperones that help determine folding (Brier, 1998; Abel, 2000; El-Hani et al., 2006; Barbieri, 2007a,b,c, 2008; Bopry, 2007; Alp, 2010; D'Onofrio et al., 2012). In addition, the mRNA sequencing of codons itself also determines the rate of translation (internal mechanism).

The external mechanisms involve trigger factors. Prokaryotes employ chaperones. Eukaryotes employ multiple chaperones, ribosome tunnel interactions, and binding proteins factors. The internal mechanisms involve mRNA interactions, codon sequences and tRNA availability (Pedersen, 1984; Andersson and Kurland, 1990; Kramer et al., 2009; Li et al., 2012). Processes that control the translation speed are called translational pausing (TP) (Li et al., 2012). They allow for momentary pauses enabling preliminary folding of the nascent protein. We will show the particular redundancy of codons provides temporal regulation of the co-translational folding process.

The focus of this paper is to show how the redundancy of the genetic code is used to prescribe functional TP. We present a brief dichotomy of the co-translational protein folding process. Once again, by co-translational, we are referring to the linked mechanisms and processes that enable and execute folding of the nascent protein *within* the domain of the ribosome. Attention also will be given to external mechanisms working in harmony with the ribosome, along with those processes working internally via the specific arrangement of nucleotides in the mRNA.

## Dichotomy of ribosomal translational folding

### Mechanistic view

Several studies suggest that ribosomes use multiple pathways to promote structural formations in nascent chains. Ribosomes can promote helix formations (Woolhead et al., 2004), compaction of arrested nascent chains (Lu and Deutsch, 2005) and possible co-translation formation of secondary and some tertiary structures (Evans et al., 2008; Kosolapov and Deutsch, 2009). In prokaryotes co-translational folding involves trigger factors and chaperones. In eukaryotes it involves primarily chaperones and binding proteins. For example the ribosome tunnel acts as a tube that can handle extended conformations and secondary structures of the peptide chain. The tunnel rim consists of RNA and ribosomal proteins. These proteins are interaction sites for ribosome-associated factors used for targeting, and folding of the peptide chain. Charge specific residues of nascent peptides slow down or stop the translation process (Kramer et al., 2009). Other pathways have been observed to regulate protein synthesis and assist with the association of factors such as the Signal Recognition Particle (SRP) (Kramer et al., 2009). Ribosomes transmit signals relating the nascent chain and its position in the tunnel to their surface, thereby controlling the interactions with SRP (Walter and Blobel, 1983; Kramer et al., 2009). The binding of SRP to the nascent protein in eukaryotes can stop the translation process (Kramer et al., 2009). Ribosomal architecture uses feedback through tunnel interactions and protein signaling to control translational folding (Marin, 2008).

Chaperones are also involved in de-novo protein folding. Chaperones work cooperatively with ribosomes in proximity to them. These co-translational activities exhibit temporal orchestration and typically act downstream in the folding process. The large number of chaperone mechanisms and their temporal interactions with nascent polypeptide chains act to coordinate co-translational folding during its growth stages. Bacterial trigger factors are ribosome associated chaperones. They work in conjunction with the nascent chains and their proximity to ribosome exit tunnels. Trigger factor interaction with the ribosome and nascent chain is a function of their length, sequence and folding status (Kaiser et al., 2006; Raine et al., 2006). By reducing the rate of folding *in vitro* and *in vivo*, trigger factors have been shown to improve the folding of model multi-domain substrates (Agashe et al., 2004). Prokaryotes use ribosome bound chaperone trigger factors. Eukaryotes use factors such as J, Hsp70, Hsp 40 and nascent chain associated complex (NAC) protein-based systems along with other such mechanisms.

Co-TP can also be induced in response to environmental stress (Liu et al., 2013). Pausing allows cells to adapt to changing environmental conditions such as heat stress. This pause has been observed where the nascent polypeptide emerges from the ribosomal exit tunnel. This has the effect of inhibiting chaperone operation by a dominant-negative mutant or other chemical inhibitors. This suggests a dual role for chaperones for both elongation and co-TP (Liu et al., 2013). Studies have shown that ribosome's can fine-tune the elongation process by sensing and reacting to the intercellular environment.

### Internal control (nucleotide arrangement)

TP of nascent proteins has also been linked to the arrangement of nucleotides in the mRNA as well as sections of nucleotide coding regions that destabilize or terminate protein synthesis. Pausing can be induced by mRNA structure (Somogyi et al., 1993), SRP binding (Lipp et al., 1987), mRNA binding proteins, rare codons (Varenne et al., 1984), and anti-Shine-Dalgarno (aSD) codon sequences (Li et al., 2012). It has been shown that replacing rare codons with more abundant codons in *Escherichia coli* or *Saccharomyces cerevisiae* has resulted in faster protein translation rates. But, these factors also adversely reduce the activity of those proteins (Crombie et al., 1992; Komar et al., 1999). This silent mutagenesis resulted in 20% lower specific activity leading to increased levels of mis-folding. Further, it has been shown that the folding efficiency of a multi-domain protein in *E. coli* has been perturbed by synonymous substitutions of rare codons by abundant tRNAs (Zhang et al., 2009). However data shows that with fixed levels of tRNA's, synonymously encoded mRNA's translate with different speeds (Sorensen et al., 1989; Sorensen and Pedersen, 1991; Li et al., 2012). For example, a silent mutation in the human gene ABCB1 caused a conformational change to occur in the P-glycoprotein. This protein folded differently caused by a temporal change in translation affecting the timing of the folding process (Kimchi-Sarfaty et al., 2007). Thus, the protein folding pathways are affected by changes in the coding regions of DNA.

An example of particle binding to the mRNA can be found in the 249 nucleotide region of c-myc mRNA known as coding region instability determinant (CRD) (Lemm and Ross, 2002). It has been hypothesized that TP occurring in the CRD region causing downstream regions of the c-myc mRNA to be susceptible to endonuclease cleavage (Lemm and Ross, 2002). This attack can occur during the pausing time unless certain binding proteins (CRD-BP) are attached to this region that shields it from the endonuclease process. The pause sites occur within the CRD c-myc region and map to rare arginine (CGA) and adjacent threonine (ACA) codons (Lemm and Ross, 2002). Data from Lemm (Lemm and Ross, 2002) shows that pause sites also occur at different codons within the CRD. The first arginine codon, however, is the strongest site. Changing both the arginine CGA and threonine ACA codon to more common synonymous codons did not cause the ribosome to pause. This supports the claim that the CGA and ACA codons are a pause site (Lemm and Ross, 2002), since CGA and ACA produce a pause, while replacing them with synonymous codons produced no pausing effect.

Recent work has built on the above observations showing a strong relationship between specific arrangements of codons in mRNA to the rate of translation (Li et al., 2012). Codon pairs within the coding regions that are similar to Shine Dalgarno (SD) sequences have shown a direct correlation to TP. In bacteria, initiation of the translation process is preceded by the acquisition of a six-nucleotide element sequence known as the SD sequence. Normally this SD sequence precedes the coding region of the mRNA transcript and allows ribosome binding at the start codon (Chen et al., 1994). The SD sequence is generally upstream of the start codon AUG (Shine and Dalgarno, 1975). The translation rate is a function of the hybridization free energy of a hexanucleotide to an aSD sequence in the 16S rRNA of the ribosome. These non-uniform rates are dependent upon embedded code (in the form of similar aSD sequences) within the body of the coding regions of the genetic message contained in the mRNA transcript (Li et al., 2012). Transient pauses have been shown to affect co-translational folding of the nascent protein by modulating the elongation process (Li et al., 2012). This temporal control plays a major role in prescribing protein functionality.

TP has been studied in very few non-bacterial species thus far. Shalgi et al. (2013) reported evidence of TP resulting in elongation pausing due to heat shock events in both mouse and human cells. Misfolding of proteins in both the cytoplasm, and during translation, triggers the cell to respond through the use of an up-regulated expression of heat shock proteins. During a heat stress event, TP is initiated in the ribosome around codon 65 in most mouse and human cells. This genome-wide phenomenon has been suggested to involve ribosome associated chaperones. Regulatory mechanisms may be involved in TP around codon 65 of most of the gene's mRNAs, resulting in elongational pausing in which a particular class of chaperones are employed to respond to heat induced misfolding (Richter et al., 2010). It still remains to be seen if codons at codon position 65 exhibit temporal tuning as a function of codon redundancy.

### Common thread between mechanistic and internal control

A common thread exists between the mechanical execution of the folding process (exit tunnel/factors/chaperones) to internal mRNA processes involved in folding of the nascent protein. We argue that the causal relationship to co-translational folding is due to a prescribed arrangement of codons within the mRNA. We base this on the fact that for trigger factors, chaperones, and binding proteins are all related to the nascent amino acid chain sequence. Amino acid sequence, by necessary consequence, points to mRNA sequences. We further posit that the interactions with translation pausing can be traced back to the specific arrangements of redundant codons in the mRNA, and ultimately to the genome. We propose that the pausing functions are facilitated by first generating a pause state in the translation of the mRNA codons within the ribosome. This gives protein factors, trigger factors and other chaperones the necessary time to mechanically perform folding operations.

“Pausing function” is caused by specific mRNA codon sequences rather than by tunnel-protein interactions to amino acid sequences. This contention is supported by data involving the substitution of rare codons with synonymous codons in *E. coli*. If the pausing effect was solely related to the amino acid chain sequence, then replacing codons with synonymous codons should still produce the same folded amino acid chain with the same translation speed. However, substitution of rare codons with synonymous codons did produce a change in speed and conformation changes (Gong and Yanofsky, 2002; Lemm and Ross, 2002; Chiba et al., 2011; Li et al., 2012).

Global analysis in bacteria indicates that 70% of strong pausing occurs when internal SD-like sequences are dominant in the coding regions (Li et al., 2012). It should be noted that canonical SD sites within the body of the coding regions are rare as opposed to low-affinity hexamers having variable rates of occurrence. This logic lines up with the hypothesis that TP causality is due to the codon sequence, which ultimately can be traced back to the genome. This cause and effect relationship provides a coherent explanation for the causality of the TP phenomenon.

Using this reasoning we have examined SD sequences in mRNA driving translation pausing within the ribosome. We examined this phenomenon to determine if a code is involved, with the inherent redundancy of the genetic code. We have examined this data in detail and will show that it exhibits the properties of a code that is used to allow for protein folding. We will show that this code resides in the same ontological prescriptive information (PI_o_) space as the genetic code used in the protein synthesis process. This dual usage of the same code within the coding regions of genes would normally be controlled semiotically if each codon had only one mapping to its corresponding amino acid. However, the genetic code is known to be redundant, meaning that multiple codons can prescribe the same amino acid. We will show that this redundancy is precisely what allows for the dual functionality of the genetic code to encode simultaneous functions within the same coding space, and using the same string of nucleotides without ambiguity. In doing so, we show why the term “degeneracy” is completely inappropriate. The dual coding functionality of redundancy is anything but “degenerate.” It represents, instead, far more sophistication, layers, and dimensions of formal prescription.

We posit that the translation pausing function is enabled by a code that is superimposed upon the genetic code, yet remains distinct and independent from the genetic code. We further posit that the genetic code consist of multi-threads of information co-existing in the same physical space which is made possible by the redundancy of the genetic code itself. To support these propositions we begin by examining the data for aSD hexamer sequences to determine the logic and rules that give it the property of code.

## Results and discussions

### Anti-shine-dalgarno translational pausing

Data used to discuss the aSD TP effect is based on two distantly related bacterial species, the Gram-negative bacterium *Escherichia coli* and the Gram-positive bacterium Bacillus subtilis (Li et al., 2012). It was thought that either the occupancy of rare tRNA's or tRNA's that are low in density population are the cause of slow translation. Authors Li, Oh and Weisssman have discovered that SD – like sequences are embedded within the mRNA coding region. These interact with the aSD site in the ribosome, and are the major causal contributors to TP (Li et al., 2012). Pausing is due to hybridization occurring at the aSD site of the 16S ribosomal rRNA and the corresponding internal SD “like” sequence contained in the coding section of mRNA (Li et al., 2012). Predicted hybridization free energy of SD like pairs of codons to the aSD sequence in the 16 rRNA section have been shown to be strong indicators of pausing (Li et al., 2012).

The data in reference Li et al. (2012) will be used extensively in this section. Authors Li, Oh, and Weissman performed data pausing analysis based on 2257 genes of *E. coli* and 1580 genes of *B. subtilis* with an average coverage of at least 10 sequencing reads per codon in the ribosome profiling data set. All of the data presented in this manuscript was taken from the Li, Oh Weissman paper and therefore takes advantage of the statistical analysis they performed on the data which is predicated on the ribosome occupancy profiles within protein coding genes. They calculated the normalized cross-correlation function for each gene with more than 10 sequencing reads per codon for samples of more than 160 base pairs in length. All of their data represented here in this manuscript is averaged over these cross correlation functions. For more information on their methods please refer to reference Li et al. (2012).

This paper defines new universal linguistic-like rules needed to identify and characterize codon mappings of TP events. This superimposes a new layer of PI_o_ on top of the traditional codon-table mappings for amino acid selection. The new rules provide a sieve through which to filter new functionality out of codon redundancy, proving that redundancy is anything but “degenerate.”

The use of these proposed filters, described accurately as formal rules, will demonstrate how codes for both amino acid selection and translation pausing can co-exist simultaneously and in the same space of contiguous sequence of nucleotides in the DNA strand. The study of such rules will aid in appreciating the far greater degree of formal sophistication, rather than degeneracy, of the genetic code. The multi-layered prescriptive information capacity of DNA sequences is vast (Duan et al., 2010; Tanizawa et al., 2010; Li et al., 2012; Stergachis et al., 2013), extending to DNA's prescription of unstructured, “disordered regions” of proteins previously thought to be inconsequential to protein function (Babu et al., 2012). The sequencing in these domains turns out to be highly prescriptive of sophisticated, integrative function operating in multiple layers and dimensions. We will confine our arguments in this manuscript, however, to just the genetic and TP code.

As the mRNA is being read by the ribosome, the speed at which the mRNA is read varies according to the certain observed codon hexamer arrangements. It has been suggested that particular hexamer sequences that enable pausing are themselves a function of a prescribed shaping needed for the elongated protein (Li et al., 2012). This reading speed modulates the folding of the nascent peptide chain, making the protein's prescribed tertiary function possible. The read speed can vary anywhere along the mRNA strand and has been observed to be a function of the affinity of hybridization between the sequences in the aSD and SD site. Hexamer sequences have been found to occur 8 to 11 nucleotides upstream of the current codon occupied in the A site. This implies that the speed information is encoded upstream of the current codon in the “A” site. The shape of the elongating protein, and translational regulation, are not only anticipated, but prescribed by upstream coding. To reiterate from a previous section, about 70% of strong pauses have been associated with internal SD like sites (Li et al., 2012).

The general consensus regarding protein synthesis centers on the idea that an mRNA prescribes a peptide sequence of amino acids for a target protein. An mRNA encoding specific proteins is one requirement. Programming those same codons to code for TP is a second requirement. Ensuring that the proposed sequences do not specify a very strong affinity for the aSD is a third requirement. Simultaneously satisfying all three programming requirements seems well beyond the capabilities of mere duplication plus variation to accomplish. Just the duality in both amino acid coding and simultaneous translation pausing coding alone must now be viewed as a minimal programming “requirement” in primordial gene emergence. Both types of prescription would have had to be incorporated from the beginning into the earliest selection of codon sequences. Additional aspects of multiple-dimension genomic prescription of biofunction are currently being elucidated. Previously, evolutionary biologists have not been aware of the conceptual complexity required for genomic programming. The required functional sequencing of codons and configurable switch-settings are far more sophisticated than ever imagined. We will address the difficulties in meeting these multiple-layered prescriptive requirements in latter sections.

### Which redundant codons prescribe TP?

There are 7 amino acids whose codonic representations are used for TP coding. They are Arginine, Glycine, Trp, Glutamic, Serine, Aspartic, and Valine (Li et al., 2012). Two combinations of codons representing a linear contiguous hexamer sequence, make up a “word” to be translated by the ribosome as it occupies the aSD site. In general the hexamer sequence will be represented in the form [X1 X2] where X1 is the primary amino acid/codon and X2 is the secondary amino acid/codon. For example, if Arginine and Glycine are used as a code for TP, we will represent the amino acid combination in the form [Arginine Glycine] and one form of its codonic representation as [AGG GGG].

### TP states and modes definitions

According to Li, Oh and Weisssman, hexamers yielding base-pairing affinities of approximately 5 or greater are considered to have substantial affinity for the aSD site (Li et al., 2012). This implies that base pairing at the aSD site with affinities greater than 5 (-kcal mol^−1^) would be candidates for stopping or reinitializing the translation process. Categorizing such hexamer sequences occurring in the coding regions as detrimental to protein synthesis implies that such states are “not allowable” (N/A). This supposition is supported by the observation that strong SD-like sequences are universally rare in *E. coli* genes (Li et al., 2012). This bias results in avoiding codon pairs that resemble canonical SD sites. A neutral state (no effect on pausing) determination is based on a pausing measure (affinity) of less than 1.0. The state of “neutral” signifies those hexamer sequences will neither pause nor terminate the translation process. They in effect have no impact on translation and are therefore neutral. The state “allowable” means that translation pausing is most likely to occur. These defined states in the DNA become modes of operation when executed by the ribosome. We now look at hexamer sequences for patterns that may be deduced as rules.

### TP data

Table 1A reorganizes the data in Figure 4 of reference Li et al. (2012) for glycine-glycine hexamer codon pair. Column 1 lists the status of the hexamer sequence and tagged as allowable, N/A or neutral in regards to its impact on enabling both the transcription and translation pausing process. The determinations of allowable and N/A are based on the pausing affinity measure of 5 (-kcal mol^−1^) or less for an allowable tag and greater than 5 for an non-allowable tag (The second column indicates the measure of the apparent affinities as generated by Li et al. (2012). Columns 3 and 4 indicate the primary and secondary codon pair situated contiguously (side by side) in both the DNA and mRNA sequence. Column 5 shows the relative rate of occurrence as observed in data of reference Li et al. (2012). The same format is used for Table 2A for arginine and glycine pairs. Data from the remaining hexamer sequences [taken from reference Li et al. (2012) as shown in reference Supplemental Figure 10] is compiled in Supplementary Tables 1a through 15a. Supplementary Tables 16a, 17a show what happens if the “N/A” threshold changes from 5 to 6(-kcal mol^−1^). It represents a crude sensitivity study for [Glycine Arginine] and [Glycine Glycine] respectively.

**Table 1A:** **Data showing pausing code (Glycine to Glycine hexamer) relative to its affinity for the aSD site**.

**Hexamer sequence**	**Affinity for aSD**	**Glycine**	**Glycine**	**Rate of occurance in mRNA**
Not allowable	(Highest affinity site) 11	GGA	GGU	Under rep Shine-Dalgarno
Not allowable	10	GGA	GGG	Under rep
Not allowable	9.6	GGA	GGA	Semi rep
Not allowable	9.2	GGG	GGU	Under rep
Not allowable	9	GGG	GGG	Under rep
Not allowable	9	GGA	GGC	Under rep
Not allowable	8.9	GGG	GGA	Semi rep
Not allowable	8.2	GGG	GGC	
Not allowable	6	GGU	GGG_2_	Semi rep
Not allowable	6	GGU	GGA	Semi rep
Not allowable	6	GGU	GGU	Semi—over rep
Not allowable	6	GGU	GGC	Semi rep
Not allowable	5.8	GGC	GGU	Over rep
Not allowable	5.6	GGC	GGG	Over rep
Not allowable	5.4	GGC	GGA	Over rep
Pause	(Least affinity site) 4.8	GGC	GGC	Over rep

*Data accumulated from reference Li et al. (2012)*.

**Table 2A:** **Data showing pausing code (Arginine to Glycine hexamer) relative to its affinity for the aSD site**.

**Hexamer sequence**	**Affinity for aSD**	**Arginine**	**Glycine**	**Rate of occurrence in mRNA**
Not allowable	8.15	AGG	GGG	Under rep Shine-Dalgarno
Not allowable	8.1	CGG	GGG	Under rep
Not allowable	7	CGA	GGU	Semi rep
Not allowable	7	AGA	GGU	Under rep
Not allowable	7	CGA	GGG	Under rep
Not allowable	7	CGA	GGA	Under rep
Not allowable	6	AGA	GGA	Semi rep
Not allowable	6	AGA	GGG	Under rep
Not allowable	7	AGG	GGA	Semi rep
Not allowable	7	AGG	GGU	Semi rep
Not allowable	5.5	CGG	GGU	Semi—over rep
Not allowable	5.5	CGA	GGC	Semi rep
Not allowable	5.5	AGA	GGC	Over rep
Pause	5	CGG	GGC	Over rep
Pause	5	CGG	GGA	
Pause	5	AGG	GGC	Over rep
Pause	2.8	CGU	GGG	
Pause	2.8	CGU	GGA	
Pause	2.5	CGC	GGA	
Pause	2.5	CGC	GGG	
Pause	2.6	CGU	GGC	
Pause	2.5	CGU	GGU	
Pause	2.5	CGC	GGC	
Pause	2.6	CGC	GGU	

*Data accumulated from reference Li et al. (2012)*.

Mode data accessed from Tables 1A, 2A have been tabulated in Tables 1B, 2B respectively for glycine and glycine designated as [glycine glycine] hexamers and [arginine glycine]. Also in Supplementary Tables 1b through 17b for the hexamer combinations of the 7 amino acid codon representations mentioned above. The table orders the hexamers relative to their affinity to couple to the aSD site. Tables 1B, 2B shows the summary of “N/A” hexamer sequence for [glycine glycine] and [arginine glycine] respectively for affinities >5.0. Supplementary Tables 1b through 15b summarize allowable and N/A hexamer sequences for other amino acid pairs with N/A affinities >5.0(-kcal mol^−1^). Supplementary Tables 16b, 17b summarize allowable and N/A hexamer sequences for N/A affinities > 6(-kcal mol^−1^).

**Table 1B:** **N/A Affinity >5.0 defined as not allowable**.

**Status**	**Glycine**	**Glycine**
Not allowable	GGA	X
Not allowable	GGG	X
Not allowable	GGU	X
Not allowable	GGC	GGU, GGG, GGA
Allowable pause	GGC	GGC

**Table 2B:** **N/A Affinity > 5.0**.

**Status**	**Arginine**	**Glycine**
Not allowable	AGG	X
Not allowable	CGG	X
Not allowable	CGA	X
Not allowable	AGA	X
Allowable	CGU	X
Allowable	CGC	X

*X means inconsequential*.

Data from Table 1A indicates that for the [gly gly] hexamer, the first codon should only be GGC and the second codon should only be GGC to enable a pause condition. Otherwise all other codon variations have affinities greater than 5 (-kcal mol^−1^) and should be avoided in the coding regions. This is summarized in Table 1B where X indicates “inconsequential,” meaning it doesn't matter which of the redundant codons for a given amino acid is used resulting in a N/A state.

Table 2A tells us that the pausing effect for the arginine glycine combination acts like a switch. When the primary codon is arginine CGU or CGC, any one of the four glycine codons will initiate a pause to the translation process. Conversely, when the primary codon is arginine AGA or CGA or CGG or AGG and the secondary codon is any one of the four possible glycine codons, this would cause a detrimental effect in the translation process and therefore, should not be selected in the DNA sequence. Our tag for this is designated N/A for “Not Allowable.” These results are captured in Table 2B. This observation of what is an acceptable codon pair, and what is not, can be formulated as a rule that could govern what is an allowable hexamer codonic pair to be used as candidates of amino acid coding in the DNA sequence of given gene.

### TP rule observation

Table 3A gives a tabulation of observations and rules that govern the primary arginine to secondary [trp, ser, arg, gly] combination with respect to pausing, neutral and N/A states for the N/A metric > 5(-kcal mol^−1^). The left most column reports the observation of states for a given hexamer defined in columns to the right. The second column from the left identifies the primary codon of the hexamer. The right two most columns identify the secondary codon. The two right most columns illustrate the dichotomy of the secondary codon. The right most column indicates only selected codons for a given amino acid will conform to the rule given in the left most column. The second right most column indicates that any of the codon representations for a given amino acid will satisfy the given rule. This acts like a binary switch behaving as all or nothing with respect to its given rule.

**Table 3A:** **Tabulation of observations and rules that govern arginine to [trp, ser, arg, gly] combination with respect to pausing, neutral, and N/A states for the N/A metric > 5**.

**Status: observation**	**Codon 1**	**Codon 2**	
**ArginineRule Stop = Affinity>5.0 N/A = Not Allowable state**		**Binary Switch Independent of codon redundancy**	**Selected Combinations**
Arginine AGG or CGG with any Gly or Trp will Produce a N/A state	AGG or CGG	{Gly or Trp}	Ser{AGU or AGC}
Arginine AGG or CGG combined with Ser(AGU or AGC) will produce a selectable N/A state			Argi{AGG or AGA}
Arginine AGC or CGG combined with Arginine (AGG or AGA) will produce a Selectable N/A State			
Arginine CGA or AGA with any GLY will cause a N/A state	CGA or AGA	GLY	
Arginine CGU or CGC combined with any Gly or Trp will produce	CGU or CGC	{Gly Trp}	
Arginine CGC or CGC combined with Arginine(AGG) will produce a selectable Pause state	CGU or CGC		Arginine{AGG}
Arginine CGC or CGU combined with Ser will produce a neutral (no pause) state	CGC or CGC	Ser	Arginine{CGC, AGA, CGC, CGA, CGG}
Arginine CGU or CGC combined with Argine (CGU, AGA, CGC, CGA, CGG) will produce a selectable neutral state			
Arginine AGA or CGA combined with Trp will produce a neutral state	AGA or CGA	Trp	Arg{AGG or CGG}
Arginine AGA or CGG combined with Arginine{AGG or CGG} will produce a selectable pause state			
Arginine AGA or CGG combined with Arginine (CGG or CGA or CGU or CGC) will produce a selectable pause state	AGG or CGG		Arginine{CGG or CGA or CGU or CGC}
Arginine AGA or CGA combined with any Ser will produce a neutral state	AGA or CGA	Ser	Arginine{CGU or AGA or CGC or CGA}
Arginine AGA combined with Arginine (CGU or AGA or CGC or CGA) will produce a selectable neutral state			
Arginine CGG or AGG combined with any ser(UCA or UCG or UCU or UCC) will produce a pause state	CGG or AGG		Ser{UCA or UCG or UCU or UCC}

The switch like action that defines the on/off (binary) state contributes to noise reduction in the Shannon channel when translated by the ribosome for pausing. For example either arginine CGA or AGA as the primary codon would cause an un-acceptable state when combined with any of the glycine codons in the secondary position of the TP hexamer.

### Rule generation

Results from Table 3A were used to generate a set of rules shown in Table 3B for generating TP states. One example of rule generation is the stipulation of what codons are allowable for an [arginine glycine] combination. The data in Table 2A assumes that pausing is necessary

Rule 1: Arginine CGU or CGC will contiguously precede glycine GGA or GGU or GGC or GGG resulting in a TP.
Rule 2: Arginine (AGG or CGG, or CGA or AGA) combined with any Glycine codon will produce a N/A state.
Rule 3: Arginine CGU or CGC when combined with Arginine CGG will produce a selectable neutral state.

**Table 3B:** **Proposed rules that govern arginine to [trp, ser, arg, gly] combination with respect to pausing, neutral, and N/A states for the N/A metric > 5**.

**Logic rules for Arginine to (Trp, Ser, Arginine, and Glysine} combinations for N/A > 5**
**Universal pausing rule:** Arginine (AGG or CGG, or CGA or AGA) combined with any Glysine codon will produce a N/A state
**Universal pausing rule:** Arginine AGG or CGG combined with any Trp will cause a N/A state
**Universal pausing rule:** Arginine AGG or CGG combined with Ser(AGU or AGC) will produce a selectable N/A state
**Universal pausing rule:** Arginine AGG or CGG combined with Arg(AGG or AGA} will produce a selectable N/A state
**Universal pausing rule:** Arginine CGU or CGC combined with Gly will produce a pause state
**Universal pausing rule:** Arginine CGU or CGC combined with Trp will produce a pause state
**Universal pausing rule:** Arginine CGU or CGC combined with Arginine AGG will produce a selectable pause state
**Universal pausing rule:** Arginine AGA or CGA combined with Trp will create a neutral state
**Universal pausing rule**: Arginine AGA or CGA when combined with Arginine AGG or CGG will produce a selectable pause state
**Universal pausing rule:** Arginine AGG or CGG when combined with Arginine CGG or CGA or CGU or CGC will produce a pausing state
**Universal pausing rule:** Arginine CGG or AGG when combined with serine UCA or UCG or UCU or UCC will produce a selectable pause state
**Universal pausing rule:** Arginine CGU or CGC or AGA or CGA when combined with Serine will produce a neutral state
**Universal pausing rule:** Arginine CGU or CGC or AGA or CGA when combined with Arginine CGC or AGA or CGU or CGA will produce a selectable neutral state
**Universal pausing rule:** Arginine CGU or CGC when combined with Arginine CGG will produce a selectable neutral state

We reiterate that by selectable, we mean that only certain codons can select for a given amino acid that would produce either a neutral, TP, or N/A state.

If the given rules conform to logical measures, then they may be well poised for functional computation via algorithmic instructions. We will examine the rational relationship of the given propositions to the logical measures of the Principles of Non-Contradiction, Identity and Excluded Middle.

We have tabulated a set of proposed rules for each of the Supplementary Tables 1b through 15b and are shown in Supplementary Tables 18–20.

### Visualization of logic measures of TP rules

**Figure 1:**
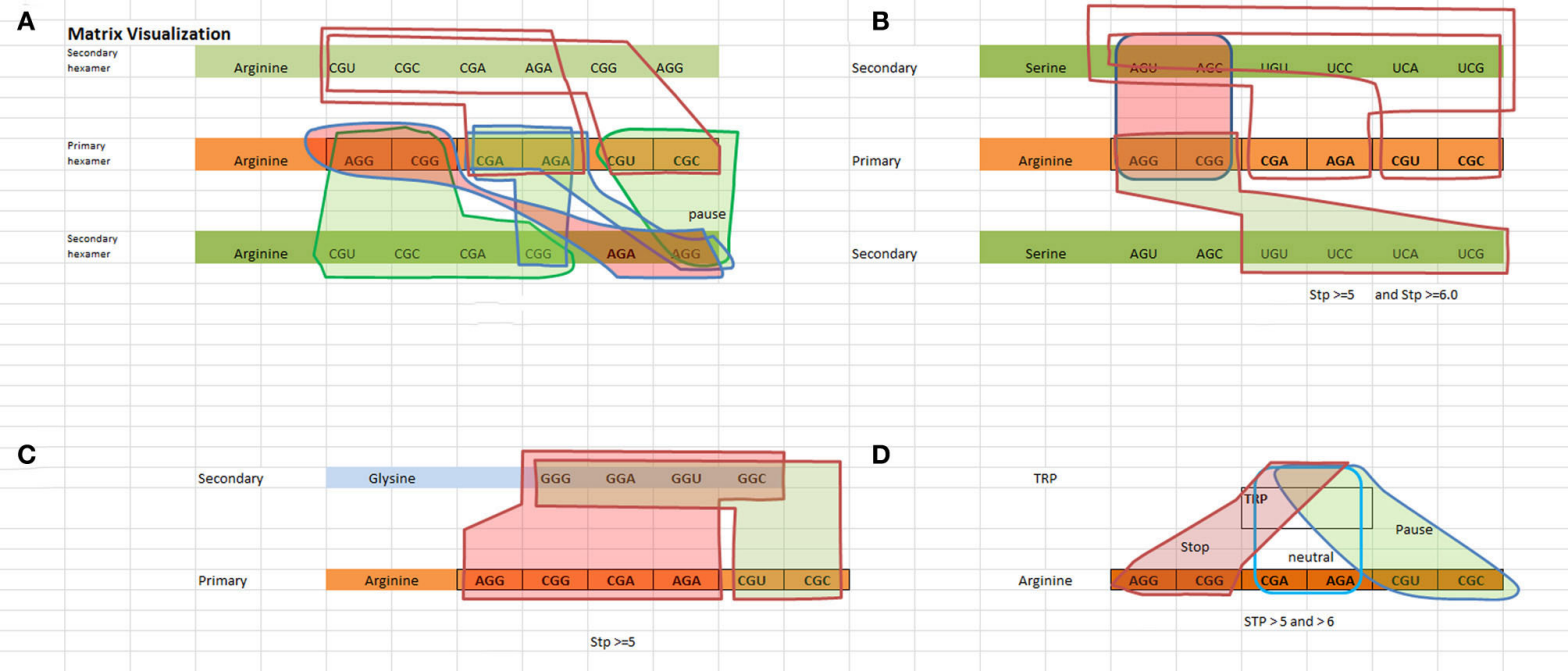
**Visualization of the TP hexamers starting with the primary codon followed by the secondary codon**. Red highlighted regions indicate a “not allowable” state, green highlighted regions indicate a “pausing” state, and no highlighted regions indicate a neutral state (no pausing). **(A)** Visualizes the States for a Arginine–Arginine hexamer. **(B)** Visualizes the states for a Arginine–Serine hexamer. Looking at the red shaded area representing “not allowed” states, any one of the red highlighted arginine codons followed by any one of the red highlighted serine codons represents a “not allowed” state. For example, [AGG ACU] or [AGG AGC] or [CGG AGU] or [CGG AGC] are not allowable. Hexamers [AGG UGU] or [AGG UCC] or [AGG UCA] or [AGG UCG] or [CGG UGU] or [CGG UCC] or [CGG UCA] or [CGG UCG] involve some level of pausing, while hexamers such as [CGA AGU, … ] represent a neutral state i.e., no pausing. **(C)** Visualizes the states for a Arginine–Glysine hexamer. **(D)** Visualizes the states for a Arginine–Trp hexamer.

We have created a way to visualize the data shown in Table 3A which is shown in Figure 1. Figure 1 illustrates the logical rules outlined in Table 3B for [Arginine Arginine], [Arginine Trp], [Arginine Glycine], and [Arginine Serine] in terms of pausing, neutral, and N/A states.

### Logic analysis

The matrix visualization of Figure 1 illustrates the unambiguous non interfering patterns of the state regions with respect to each other. The boundary conditions defined by the state regions do not cross-couple with respect to the primary codon. While the secondary codon is shared between the states, this does not void the separation of state regions. What's interesting to note is that there appears to be no ambiguity in the TP rules, i.e., rules for allowable sequences do not appear also as rules for non-allowable sequences and vice versa. It becomes readily apparent that the rules exhibit an unambiguous relationship of the primary and secondary codon structure.

1. **Identity principle**
  Within a class of state such as the “N/A” state for arginine and serine, any of the four combinations ([AGG AGU], [AGG AGC], [CGG AGU], [CGG AGC]) obey the principle of identity, i.e., all four codon pairs as shown in Figure 1B represent a class of strong canonical SD sequences, and therefore should be avoided in the coding regions. The same rationale can be inferred to the class of codons for both TP states and neutral states.
2. **Principle of excluded middle**
  The same example meets the criteria of the excluded middle. The four combinations of the non-allowable state are either non-allowable or allowable, where “allowable” means either a TP or neutral state. This defines the rule that can be included in the coding regions. With respect to the TP regions, it is true that there are varying degrees of pausing. But, all of these various temporal pausing effects are covered within the class of pausing. Therefore there is no third alternative or middle choice, i.e., the class of pausing is either pausing or it is not pausing. It cannot behave as a pause and neutral state at the same time, nor can it pause and be “N/A” at the same time.
3. **Principle of non-contradiction**
  The geometrical regions of the states shown in Figure 1 show no cross coupling and maintain separation. The data shows pausing regions cannot be either neutral or “N/A” regions. This means that “pausing regions” cannot be “non pausing regions” at the same time and in the same sense. The same applies to neutral and “N/A” regions. This satisfies the principle of non-contradiction.

It is because of both the non-interfering unambiguous state regions and the “onto” surjective functionality of the “state regions” that we can infer logical behavior to the three defined states giving them the status of logical rules. The SD TP rules behave logically and lend themselves to be compatible for computable algorithmic routines that contain nicely defined decision nodes. These can be used to calculate whether or not a pausing condition should be used in the genetic code as a function of folding. We will revisit this in the algorithm section. The SD TP rules will be shown to take on code properties that imply adherence to some level of programming language.

### Code properties

The question becomes, “Does TP exhibit code like properties?” We turn to computer science to define properties that are exhibited in computer code.

Some of the attributes of code are as follows:

1. Deterministic: The code cannot have a random structure for execution. It is deterministic in the sense that the program flow follows some type of structured flow with a certain fidelity. This does not mean it cannot have random variables. What it does mean is that an instruction and its execution is deterministic in the sense of obeying formal rules, grammar or syntax.
2. Arbitrarily chosen symbols and alphabets that are all unique in their meaning or definition.
3. Must be decodable.

There are many definitions of computer code (Code, 2012; Computer, 2013a,b) which we have condensed to the following. Code consists of a system of symbols that have arbitrarily-assigned formal meaning and function used to transfer/translate such meaning from one domain to another.

Computer code consists of the symbolic arrangement of data and or instructions that follow rules and or imposed formal structure. These rules may be a form of grammar or syntax imposed on both data and instruction. The set of rules that defines the flow of instructions and data transfer arbitrarily-assigned formal meaning and function from one domain to another.

By data, we mean:

Data is an abstract representation of either contrived meaning or reality using symbol sequences that collectively prescribe meaning and/or computational function.

In the sense of genetic code, data is not the physical interactions of phenomena or the phenomena themselves. It is therefore important to differentiate data from actual physical phenomena. The selection of nucleoside bases (A, C, T, G, U) is similar to the selection of physical tokens when playing the game of Scrabble. This is referred to as a Material Symbol System (MSS) (Rocha, 2000, 2001). Each selection of a nucleoside also serves as a formalistic configurable switch-setting. Each nucleoside selection serves to instantiate programming choices into physical reality from the non-physical formal world. Using these definitions we see that the proposed TP hexanucleotide sequence is analogous to a Word composed of the genetic alphabet consisting of the letters (A, C, G, T, U). These words have been shown to have non ambiguous and deterministic meaning adhering to strict syntax or grammar structure as defined in the “TP Rule observation” and “Rule generation” sections above.

The hexanucleotide word definition defined in the coding section of a gene conveys the temporal pausing data from the genome domain to the protein domain via decoding algorithms instantiated into hardware within the ribosome. Thus arbitrarily encoded data in the genome having formal meaning and function is transmitted from the genome domain to the protein domain and decoded to explicitly convey temporal pausing information to the ribosome. We therefore conclude that the TP codons in the genome constitute a code co-existing with the genetic code.

### Link to machine language

In computers, machine code in the form of machine language consists of a set of instructions or data executed by a computer's central processing unit (CPU). Instructions are represented as patterns of binary bits that are recognized by the CPU machine to perform physical operations. There must not be any ambiguity in the mapping of bit patterns to physical operations of the CPU. Such ambiguous instructions could interrupt the instruction execution flow (program flow) or result in faulty command recognition causing inconsistent results. Ensuring that the mappings are logically coherent can ensure that the language encoded in the bit pattern of the instructions is unambiguously interpreted by the CPU machine.

In a likewise manor, the coding regions of genes consist of bit patterns that can also be representative of commands used by a biological machine, such as the ribosome. We show in this paper that the bit patterns representing TP instructions follow logical and linguistic rules that support their use in a non-ambiguous way. The patterns organized in the TP code are strongly associated with the architecture of the ribosome. If this were not true, then inductively we would not expect the ribosome to coherently control the pausing time resulting in erratic folding.

### Dual function TP/protein prescription analysis

Chronologically and causally, “meaning” is *first* contained in the codonic prescriptive sequence, which then instructs amino acid (AA) sequencing and TP (Brier, 1998; Abel, 2000; El-Hani et al., 2006; Barbieri, 2007a,b,c, 2008; Bopry, 2007; Alp, 2010; D'Onofrio et al., 2012). The sequence of specific R groups determines the minimum-free-energy folding of the protein (Muff and Caflisch, 2009; Schuetz et al., 2010; Liwo et al., 2011; Contessoto et al., 2013; Marinelli, 2013). Thus, the prescribed sequencing blossoms into deeper layers of meaning (Abel, 2000, 2011c, 2012a,b; Kellera, 2011; D'Onofrio et al., 2012). In molecular biological messages, “meaning” translates into successful “biofunction” (Huttegger, 2007; Kauffman, 2007; Kolen and Van de Vijver, 2007; Livaditis and Tsatalmpasidou, 2007; Sherman and Deacon, 2007; Abel, 2009c; Marsen, 2009; Alp, 2010; Bedau, 2010; Benner, 2010; Levy, 2010; Montañez et al., 2010; Tirard et al., 2010).

The phenomenon of TP presents unique challenges in compressing multiple messages co-incident within the same nucleotide sequence residing in the ORF of the given gene element. *Clearly, two distinct messages are instantiated into the same codon sequence of “tokens.”* One instructs TP; the other instructs protein prescription. Both reside in the same token sequence space within the DNA gene element (ORF). For these messages to simultaneously occupy the same sequence space and yet remain orthogonal in terms of their functionality means that the configuration or sequencing of nucleotides have multiple and independent meanings and functions. In general it is hypothesized that the holistic protein synthesis function can be decomposed into “n” independent sets of formal function prescriptions. Each is found in a different layer of PI_o_. One layer is superimposed onto the other. The corresponding wetware of DNA and the cell allows deciphering of independent messages.

**Figure 2:**
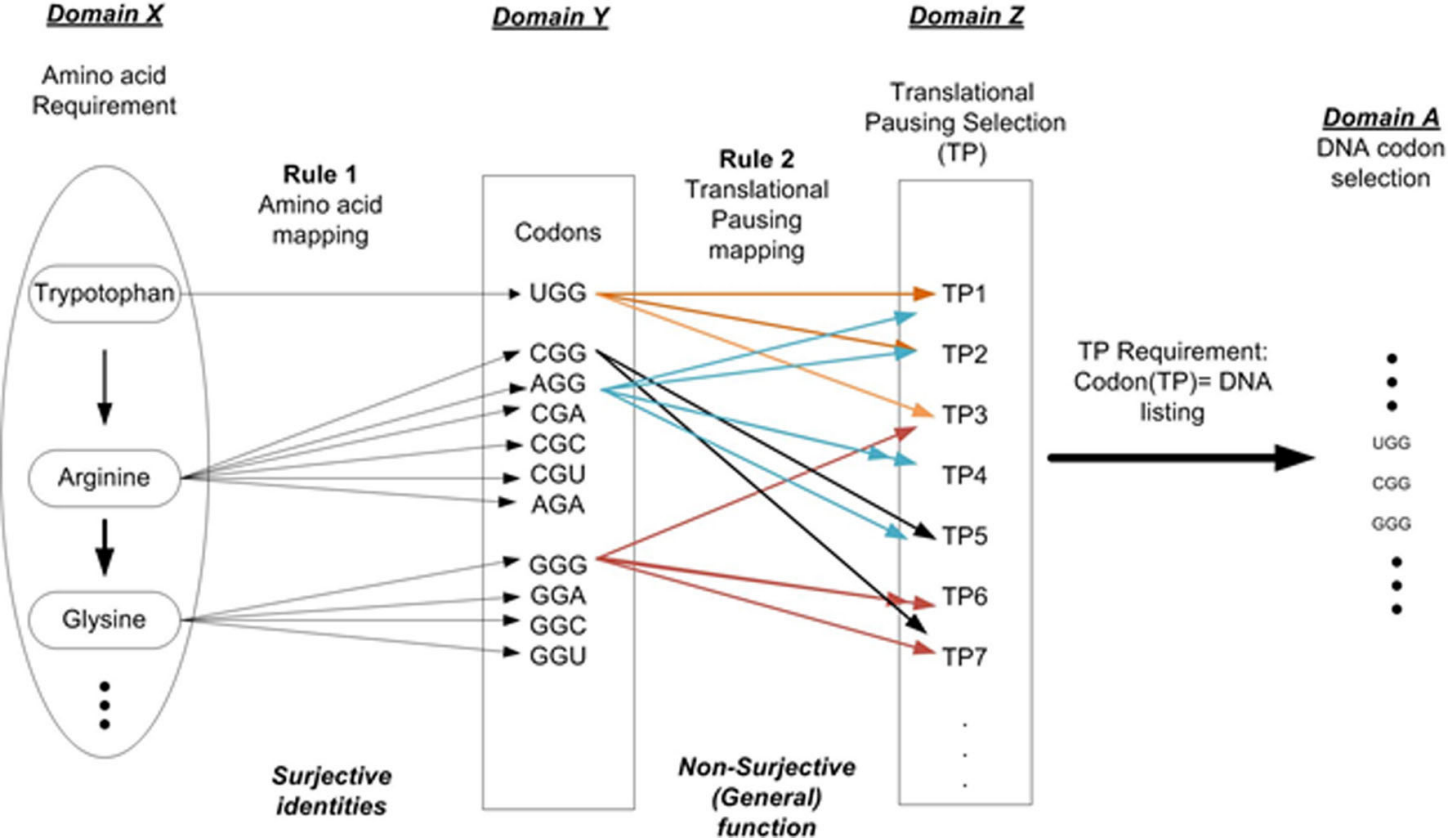
**DNA codon selection that illustrates the multi-layer requirements**. Requirement 1: Amino acid selection to prescribe protein requirement. Requirement 2: Folding requirement in terms of the pausing of the translation process.

For example setting *n* = 2 allows us to describe two independent layers of instructions as shown in Figure 2. Layer 1 is the linear sequential prescription of amino acids that defines the protein's primary structure (amino acid sequencing). This is represented as Domain X. Rule 1 (amino acid mapping) is applied to identify all codons for a given AA and is shown in Domain Y. Layer 2 is the TP sequence symbolically representing information necessary to modulate protein folding during the elongation process. Rule 2 defines the various TP commands as a function of Domain Y. This result is represented as Domain Z, the set of TP commands. Given a TP requirement results in the final selection of codons for a given protein prescription and is shown in Domain A. The superposition of layers 1 and 2 on the DNA strand contributes to the protein synthesis process. This must occur within the same nucleotide sequence space of the ORF gene element without interfering with each other. The arrangement of nucleotides that code the sequential arrangement of amino acids appears as prescribed data to the embedded algorithm i.e., mechanisms of the ribosome (D'Onofrio et al., 2012). The arrangement of nucleotides that define the message of TP appears as another set of formal instructions and controls (not mere physical constraints). We must remember constantly that the nucleotides are tokens in a MSS (Rocha, 2001; Abel, 2011b). Sequencing is arbitrarily selected and rule-based, free from the constraints of initial conditions and law.

### Multi-threading

In computer science, a thread is the smallest sequence of programmed instructions that an operating system scheduler can manage independently. Multi-threading is a widespread executing and programming model that allows multiple threads to co-exist within the context of a single process. Multi-threads are able to share the resources of a given process while executing concurrently. We posit that the operation of the ribosome can be viewed as a type of physical multi-core processor in terms of concurrently executing amino acid elongation and pausing control to enable protein folding. Within the context of the protein synthesis process, we posit that the two independent threads of information co-exist within the same nucleotide sequence because of the redundancy of the genetic code as shown in the previous sections.

Specifically, having multiple codon codes prescribing the same amino acid allows any one of those redundant codonic prescriptions to have an alternate coded meaning which can produce a completely different biofunction. For example, Glycine can be represented by any one of the following four codons: GGA, GGT, GGA, and GGC. Any one of these codons represented in the DNA ORF will be interpreted as the amino acid Glycine by the ribosome (thread 1). Because of the contingency of the glycine representative codon code, we can assign alternative functions to any four of the four codons (thread 2). Hypothetically, we could assign four different translation speeds such as speed 1 to GGT, speed 2 to GGC speed 3 to GGG and ignore speed change to GGA.

### Independent decoding ciphers and processes are needed for each layer of instruction

In conventional computers, multi-threaded code is written using the same machine language. When only a single processor is used, the code is written in nested form allowing a scheduler to determine when parts of the nested code are to be executed. Only one nested thread can be executed at a time. Thus, multiple threads are executed in series scheduled one thread at a time. This permits multi-threaded computation. Using multi-cores (multiple CPU's), the code must be written such that the scheduler can parse the code to each CPU for parallel execution. However, our biological system uses the same nucleotide tokens residing in the same space that contain multiple meanings. We want to distinguish and emphasize here that we are not talking about nested code, but *coincident code*, i.e., using the same string of bits for two different translations. Deductively, these messages cannot use the same language because they occupy the same space and tokens (bases). Different languages or mappings must be used to distinguish the two threads. In order to interpret these co-existing multi-threaded languages, there must be two independent decoding mechanisms (multi-cores) that can read and decipher the linear sequence of codons carried by the mRNA. Such a mechanism must be synchronized with the same starting point of both messages. This places a further constraint on the control methodologies used to synchronize the start and end points of both independent messages in the DNA. Having the two messages out of sync with each other would be analogous to an out-of-frame reading error. The way in which the prescriptive information space is compressed and utilized may represent an optimized approach of data compression. This represents a departure from the conventional way multi-threading is done in the computer world.

We now posit a model that explains the ribosome behavior regarding the decomposition of the genetic code presented in the input mRNA. The ribosome functions as a multi core processing protein synthesis machine. It is multi core in the sense that it can simultaneously process two threads of encoded information independently as discussed in the Multi-thread section above.

Core A is defined as the machinery governed by the rules of codon to amino acid mapping. These mappings are determined by the tRNA definitions which themselves are governed by rules outside of the ribosome. The tRNA's are part of a formal “system” going on somehow in the cell that organizes and employs tRNAs to accomplish function that is simply transcendent to physicality and the tRNA tokens themselves. The synthesis function is determined by the PI_o_ embodied in the ribosomal computer-mediated independent machinery, and its contribution to formal “systems biology.” This consists of, but is not limited to, *Ports* A, B and C, codon advancer, and amino acid binding function.

*Core* B is the hardware internal to the ribosome in the form of the aSD site that interprets both protein synthesis initiation and translation speed/pausing thread relative to the thread of *Core A*. These two cores operate independently of each other, meaning that there is no communication or feedback between these processes (i.e., Core A isn't influenced by operations in Core B, and vice versa). Despite this, the ribosome acts holistically in concert with the protein synthesis process to produce a prescribed nascent protein. Since the two threads work independently and blindly with respect to the nascent protein, this suggests that the information to co-actively synchronize and coherently adjudicate these two threads must originate outside of the ribosome.

The duality in the coding function acts to remove the redundancy in the genetic code when viewed holistically. We now posit a model of the how multi-dimensional information consisting of both translation pausing time data and DNA to amino acid mappings could be accomplished.

### Execution of the multi-dimensional (genetic code/TP) algorithmic approach

We now present a scenario of how the of the DNA ORF sequence could be synthesized based on two-specifications, i.e., amino acid sequence and nascent time-based transcriptional pausing. The requirements in this example below are assumed to be known *a priori*.

**Figure 3:**
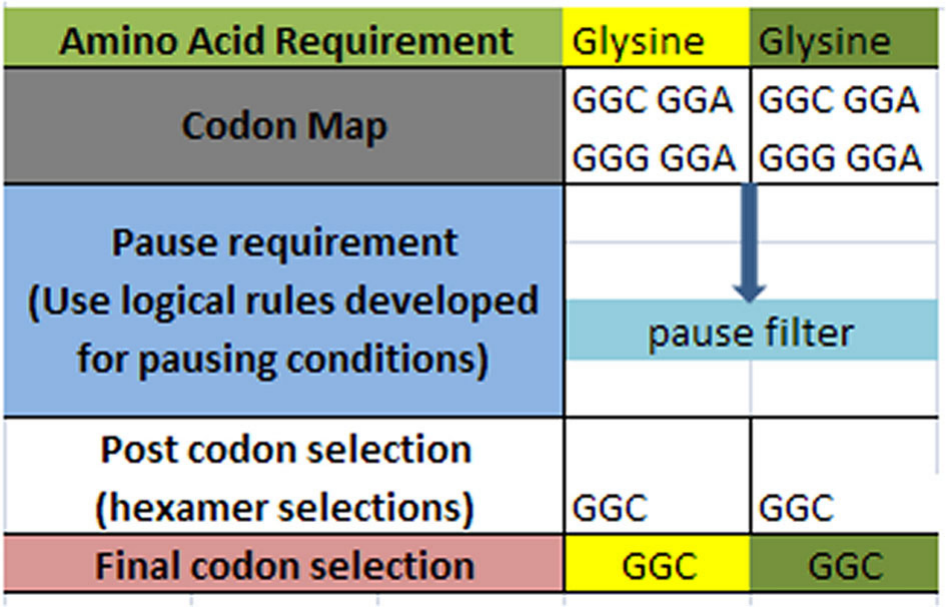
**Hypothetical amino acid/TP selection example**. Top row requires the given partial amino acid series for a given protein (left to right). The second row specifies the standard codon map for its particular amino acid. The pause requirement row specifies that a translation pause is necessary for the glycine to glycine junction for proper pre-folding. The rules for pausing are invoked to filter the redundant glycine codons. The post codon selection row results from filtering the codon map above for specified TP condition. The final codon selection row is the final codon selection from the row immediately above (may or may not be a function of other filters). This results in writing the ORF of a hypothetical gene.

A simple example of the selection process is shown in Figure 3 below for a simple glycine to glycine sequence. It is required that the glycine amino acid must follow the current glycine amino acid in our simple ORF example. In addition, it is required that the [glycine_glycine] part be allowed to pause in the ribosome chamber for a set amount of time to allow for preliminary folding of the [glycine_glycine] pair. The DNA sequence process would begin by selecting the codon representations for both the glycine-glycine pair. Next, the process would filter the codon sets for these two contiguous representations for those codons that meet the timing (pause) requirement. Filtering through the redundant codons results in a selection of candidate codons as shown in row four (post codon selection). In general there could exist additional filters which could further discriminate the post codon selection. Finally, the resultant codon is written into the DNA ORF sequence as shown in row 5.

**Figure 4:**
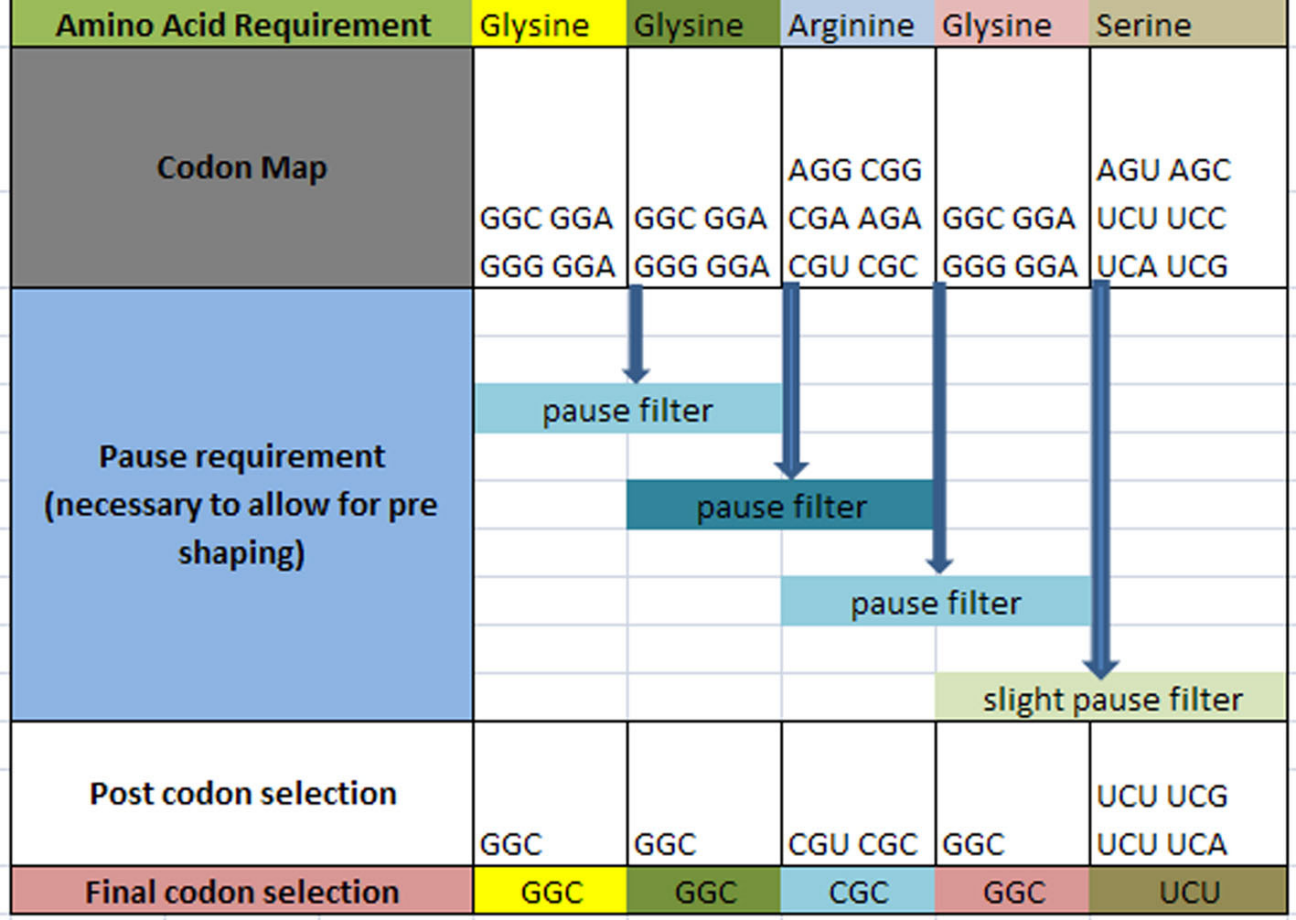
**Hypothetical amino acid/TP selection series example**. Top row requires the given partial amino acid series for a given protein (left to right). The second row specifies the standard codon map for its particular amino acid. The pause requirement row specifies that a translation pause is necessary for the glycine to glycine junction, the glycine to arginine junction, arginine to glycine junction, and glycine to serine junction for proper pre-folding. The post codon selection row results from filtering the codon map above for specified TP condition. The final codon selection row is the final codon selection from the row immediately above (may or may not be a function of other filters). This results in writing the ORF of a hypothetical gene.

The question arises as to how codon selection would precede in a continuous chain of a prescribed amino acid representation for a given protein. Figure 4 illustrates the selection process for such a case. In this hypothetical case, it is required that within the ORF of a hypothetical protein, the following amino acids are to be sequenced with the following requirement: … Glycine_1_ Glycine_2_ Arginine_3_ Glycine_4_ Serine_5_ … in a consecutive order as shown in row 1 (the subscript denotes the sequential amino acid position within the ORF). The codons for each amino acid are assembled and shown in row 2. A requirement exists for a set amount of pausing for each amino acid boundary defined as [glycine_1_ glycine_2_] followed by [glycine_2_ arginine_3_] followed by [arginine_3_ glycine_4_] and [glycine_4_ serine_5_] as shown on row 3. Each boundary condition is subject to the logical rules that define the amount of pausing of the translation process and is illustrated as a “pause filter” for that boundary condition. The result of filtering the codon map of row 2 results in a subset of codons shown in row 4. Notice that the selection of the codon for glycine2 is dependent on the next boundary condition immediately following the current boundary condition. This dependency establishes an iterative selection process. A codon pair that meets the current boundary condition may not reside in the solution space for the next boundary condition. In other words, the selection of the hexamer sequence for a given boundary condition must be the prerequisite condition for the next boundary condition. This becomes a constraint in our selection process that must be accounted for. This results in an iterative process to successfully filter this chain of code. Finally, allowing for additional filtering effects, undefined here are shown in row 4, the final selection of codons is written into the DNA ORF.

### TP and pathological consequences

We posit that the TP effect allows time for upstream mechanisms to control the folding process of the elongating protein. The regulation processes correct for misfolding due to external environmental effects. Heat stress is a prime example. Examples of the upstream products were discussed in the previous section Mechanistic view. They include in eukaryotes, chaperones, binding proteins and tunnel interactions with ribosome associated factors. This ribosome architecture works in conjunction with protein signaling feedback to control translational folding. In prokaryotes, co-translational folding involves trigger factors and chaperones that are coupled to codon mappings that initiate TP allowing the up-stream folding process to function. We posit that misfolding of the nascent protein or un-regulated chaperone could result without proper TP functionality.

Point mutations, and their effects on redundant codons, can be seen when such mutations affect the timing pause as dictated by the rules we posit for TP pausing. For example a point mutation of the Arginine codon from CGG to AGG followed by an un-mutated Glycine GGA codon would likely stall or terminate the translation process. This would occur without affecting the proper amino acid prescription.

## Conclusion

Redundancy in the primary genetic code allows for additional independent codes. Coupled with the appropriate interpreters and algorithmic processors, multiple dimensions of meaning, and function can be instantiated into the same codon string. We have shown a secondary code superimposed upon the primary codonic prescription of amino acid sequence in proteins. Dual interpretations enable the assembly of the protein's primary structure while enabling additional folding controls via pausing of the translation process. TP provides for temporal control of the translation process allowing the nascent protein to fold appropriately as per its defined function. This duality in the coding function acts to reduce the redundancy in the genetic code when viewed holistically. The functionality of condonic redundancy denies the ill-advised label of “degeneracy.” When simultaneously combined with other coding schemas such as intron/exon boundary conditions, and overlapping and oppositely oriented promoters, multiple dimensions of independent coding by the same codon string has become apparent.

The ribosome can be thought of as an autonomous functional processor of data that it sees at its input. This data has been shown to be PI_o_ in the form of prescribed data (D'Onofrio et al., 2012), not just probabilistic combinatorial data. Choices must be made with intent to select the best branch of each bifurcation point, in advance of computational halting.

The arrangement of codons has embodied in it a prescribed sequential series of both amino acid code and time-based TP code necessary for protein assembly and nascent pre-folding that defines protein functionality. We have shown that the TP coding schema follows distinct and consistent rules. We have demonstrated that these rules are logical and unambiguous. The actual hexanucleotide “word” selection is dependent upon the next adjacent codon. This conditional selection is shown in the algorithm section. Such an iterative process nicely lends itself to an algorithmic process should geneticists experiment with writing their own genetic code. Understanding the dual mappings between the amino acid and TP code will allow algorithmically computed solutions to simultaneously fulfilling the this dual requirement using the same written code.

It has been shown that both the genetic code and TP code are decoupled allowing simultaneous decoding and dual functionality within the ribosome using the same alphabet (nucleotides) but different languages. With other languages such as French, we share the same alphabet, but employ different semantic and grammatical rules. The same is true of the codon alphabet being used by the cell to generate more than one language.

The TP code exhibits distinct meaning in relation to mappings between codons and pausing units. The TP code also exhibits a syntax or grammar that obeys strict codon relationships that demonstrate language properties. Because of the redundancy of the genetic code, it could be argued that the TP language is a subset of the genetic language. The subspace of the TP language resides, and thus appears to have a dependency on, the primary genetic code. Within this subspace, however, we argue that the TP language is decoupled from and remains independent of the protein-coding language.

Hypothetically, in a non-redundant codon to amino acid mapping, once the codon sequence is selected, thereby defining the prescribed amino acid chain, this prescription would preclude additional information from occupying the same space (ORF) to prescribe TP. The only way the physical constraints could be removed from formal PI_o_ instantiation of additional TP controls using the same code would be to build redundancy into the genetic language. Thus, having redundant contingency in the genetic code is both a necessary and sufficient condition to represent multiple languages using the same alphabet of the genetic code.

Amino acid sequence, by necessary consequence, points to mRNA sequences. We further posit that the interactions with translation pausing can be traced back to the specific arrangements of redundant codons in the mRNA, and ultimately to the genome. We propose that the pausing functions are facilitated by first generating a pause state in the translation of the mRNA codons within the ribosome. This gives protein factors, trigger factors and other chaperones the necessary time to mechanically perform folding operations.

More research is needed to determine a higher level of fidelity in regards to specific timing of pauses relative to TP codons and nascent folding in order to understand its impact on disease. According to Shalgi et al. (2013), misfolding of the nascent protein can lead to certain cancers. Misfolding stress, and the heat shock response pathway in particular, play specific developmental roles, and are implicated in a variety of diseases. Upregulation of chaperones is frequently observed in cancer. Chaperone inhibitors hold promise as antitumor agent (Whitesell and Lindquist, 2005; Calderwood et al., 2006). Overexpression of eukaryotic proteins with strong internal SD sites would sequester ribosomes and compromise protein yield (Li et al., 2012).

## Author contributions

David J. D'Onofrio conceived the overall concept including generation of figures and tables. David J. D'Onofrio managed iterative refinement of this review from input provided by co-author. David L. Abel provided major insight into subject matter contributing to the technical content and refinement of the manuscript. All authors contributed to writing the manuscript.

### Conflict of interest statement

The authors declare that the research was conducted in the absence of any commercial or financial relationships that could be construed as a potential conflict of interest.

## Abbreviations

PI_o_: Ontological Prescriptive Information
MSS: Material Symbol System
FI: Functional Information
TP: Transcriptional Pausing
DI: Descriptive Information
AA: Amino acid
SD: Shine Dalgarno sequences
aSD: anti Shine Dalgarno sequence.
