# Supplementary material for: Redundancy of the genetic code enables translational pausing
Source: Front Genet. 2014 May 20;5:140. doi: 10.3389/fgene.2014.00140 (PMC4033003; doi:10.3389/fgene.2014.00140)
Supplement: Supplementary file 1 [file DataSheet1.DOCX]

**Supplementary Section: Data and Graphs**

**Supplementary Table 1a** Data showing pausing code (Tryptophan to Glycine hexamer) relative to its affinity for the aSD site. Data accumulated from ref [[30](#_ENREF_30)].

| Status | Trp | Gly |
| --- | --- | --- |
| Not Allowable | UGG | GGG, GGU, GGA |
| Allowable | UGG | GGC |

**Supplementary Table 1b** N/A Affinity >5.0 X means inconsequential

 **Supplementary Table 2a**  Data showing pausing code (Glutamic to Glycine hexamer) relative to its affinity for the aSD site. Data accumulated from ref [[30](#_ENREF_30)].

| **Status** | **Glutamic** | **Glysine** |
| --- | --- | --- |
| Not Allowable sequence | GAG | X |
| Pause | GAA | X |

**Supplementary Table 2b** N/A Affinity >5.0 X means inconsequential

 **Supplementary Table 3a**  Data showing pausing code (Glysine to Glucose hexamer) relative to its affinity for the aSD site. Data accumulated from ref [[30](#_ENREF_30)].

| Status | Glycine | Glutamic |
| --- | --- | --- |
| Not Allowable sequences | GGA | GAG, GAA |
| Not Allowable sequences | GGG | GAG, GAA |
| Not Allowable sequences | GUU | GAG, GAA |
| Not Allowable sequences |  |  |
| Pausing | GGC | GAG, GAA |
|  |  |  |

**Supplementary Table 3b** N/A Affinity > 5.0 X means inconsequential

**Supplementary Table 4a**  Data showing pausing code (Glysine to Serine hexamer) relative to its affinity for the aSD site. Data accumulated from ref [[30](#_ENREF_30)].

| Status | Glycine | Serine |
| --- | --- | --- |
| Not Allowable sequences | GGG | AGU, AGC |
| Pause | GGA | X |
| Pause | GGU | X |
| Pause | GGG | UCG, UCA, UCU, UCC |
| Pause | GGC | X |
|  |  |  |

**Supplementary Table 4b** N/A Affinity >= 5.0 X means inconsequential

 **Supplementary Table 5a**  Data showing pausing code (Glysine to Valine hexamer) relative to its affinity for the aSD site. Data accumulated from ref [[30](#_ENREF_30)].

| Status | Glycine | Valine |
| --- | --- | --- |
| Not Allowable sequences | GGG | X |
| Not Allowable sequences | GGA | X |
| Not Allowable sequences | GGU | X |
| Pause | GGC | x |
|  |  |  |

**Supplementary Table 5b** N/A Affinity >5.0 X means inconsequential

 **Supplementary Table 6a**  Data showing pausing code (Glysine to Asperine hexamer) relative to its affinity for the aSD site. Data accumulated from ref [[30](#_ENREF_30)].

| Status | Glycine | Asp |
| --- | --- | --- |
| Not Allowable sequences | GGG | X |
| Not Allowable sequences | GGA | X |
| Not Allowable sequences | GGU | X |
| Pause | GGC | x |
|  |  |  |

**Supplementary Table 6b** N/A Affinity > 5.0 X means inconsequential

 **Supplementary Table 7a**  Data showing pausing code (Glycine to Tryptophan hexamer) relative to its affinity for the aSD site. Data accumulated from ref [[30](#_ENREF_30)].

| Status | Glycine | Tryptophan |
| --- | --- | --- |
| Not Allowable sequences | GGG | X |
| Not Allowable sequences | GGA | X |
| Pause | GGU | X |
| Pause | GGC | x |
|  |  |  |

**Supplementary Table 7b** N/A Affinity > 5.0 X means inconsequential

 **Supplementary Table 8a**  Data showing pausing code (Arginine to Arginine hexamer) relative to its affinity for the aSD site. Data accumulated from ref [[30](#_ENREF_30)].

| Status | Arginine | Arginine |
| --- | --- | --- |
| Not Allowable sequences | AGG | AGG,AGA |
|  | CGG | AGG,AGA |
| Allowable | AGA | AGG, CGG |
| Allowable | AGG | CGG, CGA, CGU, CGC |
| Allowable | CGG | CGG, CGA, CGU, CGC |
| Allowable | CGU | AGG |
| Allowable | CGC | AGG |
| Allowable | CGA | AGG, CGG |
| Neutral (no pausing) | AGA | CGU, AGA, CGC, CGA |
| Neutral (no pausing) | CGU | CGU, AGA, CGC, CGA, CGG |
| Neutral (no pausing) | CGC | CGU, AGA, CGC ,CGA, CGG |
| Neutral (no pausing) | CGA | CGU, AGA, CGC, CGA |

**Supplementary Table 8b** N/A Affinity > 5.0 X means inconsequential

 **Supplementary Table 9a**  Data showing pausing code (Arginine to Tryptophne hexamer) relative to its affinity for the aSD site. Data accumulated from ref [[30](#_ENREF_30)].

| Status | Arginine | Tryptophan |
| --- | --- | --- |
| Not Allowable sequences | AGG | X |
| Not Allowable | CGG | X |
| Allowable | AGA | X |
| Allowable | CGA | X |
| Allowable | CGU | X |
| Allowable | CGC | X |

**Supplementary Table 9b** N/A Affinity > 5.0 X means inconsequential

 **Supplementary Table 10a**  Data showing pausing code (Arginine to Serine hexamer) relative to its affinity for the aSD site. Data accumulated from ref [[30](#_ENREF_30)].

| Status | Arginine | Serine |
| --- | --- | --- |
| Not Allowable sequences | AGG | AGU |
| Not Allowable sequences | CGG | AGU |
| Not Allowable sequences | AGG | AGC |
| Not Allowable sequences | CGG | AGC |
| Pausing | CGG | UCA, UCG, UCU, UCC |
|  | AGG | UCU, UCA, UCG, UCC |
|  |  |  |
| Neutral (no pausing) | AGA | X |
| Neutral (no pausing) | CGU | X |
| Neutral (no pausing) | CGC | X |
| Neutral (no pausing) | CGA | X |

**Supplementary Table 10b** N/A Affinity > 5.0 X means inconsequential

 **Supplementary Table 11a**  Data showing pausing code (Tryptophne to Arginine hexamer) relative to its affinity for the aSD site. Data accumulated from ref [[30](#_ENREF_30)].

| Status | Tryptophan | Arginine |
| --- | --- | --- |
| Not Allowable sequences | UGG | AGG, AGA |
| Allowable | UGG | CGG, CCA, CGU, CGC |

**Supplementary Table 11b** N/A Affinity > 5.0 X means inconsequential

 **Supplementary Table 12a**  Data showing pausing code (Glycine to Tryptophan hexamer) relative to its affinity for the aSD site. Data accumulated from ref [[30](#_ENREF_30)].

| Status | Glycine | Tryptophan |
| --- | --- | --- |
| Not Allowable sequences | GGG | UGG |
| Not Allowable sequences | GGA | UGG |
| Pause | GGU | UGG |
| Pause | GGC | UGG |

**Supplementary Table 12b** N/A Affinity > 5.0 X means inconsequential

 **Supplementary Table 13a**  Data showing pausing code (Glutamic to Aspartic hexamer) relative to its affinity for the aSD site. Data accumulated from ref [[30](#_ENREF_30)].

| Status | Gluutamic | Aspartic |
| --- | --- | --- |
| Not Allowable sequences | GAG | X |
| Neutral | GAA | X |

**Supplementary Table 13b** N/A Affinity >= 5.0 X means inconsequential

 **Supplementary Table 14a**  Data showing pausing code (Glutamic to Glutamic) relative to its affinity for the aSD site. Data accumulated from ref [[30](#_ENREF_30)].

| Status | Glutamic | Glutamic |
| --- | --- | --- |
| Not Allowable sequences | GAG | X |
| Pause | GAA | GAG |
| Neutral | GAA | GAA |

**Supplementary Table 14b** N/A Affinity > 5.0 X means inconsequential

 **Supplementary Table 15a**  Data showing pausing code (Glutamic to Valine hexamer) relative to its affinity for the aSD site. Data accumulated from ref [[30](#_ENREF_30)].

| Status | Glutamic | Valine |
| --- | --- | --- |
| Not Allowable sequences | GAG | X |
| Pause | GAA | GUG, GUA |
| Neutral | GAA | GUC, GUU |

**Supplementary Table 15b** N/A Affinity > 5.0 X means inconsequential

**Supplementary Table 16a**  Sensitivity study. Data showing pausing code (Glycine to Arginine hexamer) relative to its affinity for the aSD site. N/A site >= 6.0. Data accumulated from ref [[30](#_ENREF_30)].

| Status | Glycine | Arginine |
| --- | --- | --- |
| Not Allowable sequences | GGG | AGG, AGA |
| Not Allowable sequences | GGA | CGG, AGG |
| Not Allowable sequences | GGU | AGG |
| Not Allowable sequences | GGC | AGG |
|  |  |  |
| Pausing | GGG | CGG, CGU, CGA, CGC |
| Pausing | GGA | CGU, CGA, AGA, CGC |
| Pausing | GGU | AGA, CGU, CGC, CGG, CGA |
| Pausing | GGC | AGA, CGU, CGC, CGG, CGA |

**Supplementary Table 16b** Sensitivity study. N/A Affinity >6.0 X means inconsequential

 **Supplementary Table 17a**  Sensitivity study. Data showing pausing code (Glycine to Glutamic hexamer) relative to its affinity for the aSD. N/A site >= 6.0. Data accumulated from ref [[30](#_ENREF_30)].

| Status | Glycine | Glutamic |
| --- | --- | --- |
| Not Allowable sequences* | GGA | GAA, GAG |
| Not Allowable sequences* | GGG | GAG |
| Not Allowable sequences |  |  |
| Not Allowable sequences |  |  |
|  |  |  |
| Pausing | GGU | GAG, GAA |
| Pausing | GGG | GAA |
| Pausing | GGC | GAG, GAA |

**Supplementary Table 17b** Sensitivity study. N/A Affinity >=6.0 X means inconsequential

| **Pause Law:** Trp combined with Gly(GGG or GGA or GGU) codon is a selectableN/A state  **Pausing Law:** Trp combined with Arginine(AGG or AGA) codon is a selectableN/A state.  **Pausing Law:** Trp combined with Gly GGC codon is a selectablepause state.  **Pausing Law:** Trp combined with Arginine (CGG or CCA or CGU or CGC) is a pausing state. |
| --- |

**Supplementary Table 18** Logic Laws for Trp as primary codon

| **Pausing Law:** Glutamic GAG combined with any Gly, Asp, Glu or Val codon creates a N/A state  **Pausing Law:** Glutamic GAA combined with any Gly, Glu, or Asp codon will create a pause state  **Pausing Law:** Glutamic GAA combined with Val{GUG or GUA} will produce a selectable pause state.  **Pausing Law:** Glutamic GAA combined with Val {GUC or GUU} will be neutral to translation (Neutral) |
| --- |

**Supplementary Table 19** Logic Laws for Glutamic as primary codon

| **Pausing Law:** Glycine {GGG, GGA} combined with any Gly or Glu or Val or Asp or Trp creates a stop state.  **Pausing Law:** Glycine (GGU ) combined with any Ser or Trp will produce a pause state.  **Pausing Law:** Glycine (GGC) combined with any Asp, Trp, Val, Ser, or Glu will produce a pause state.  **Pausing Law:** Glycine GGC, GGA or GGU combined with Arginine (AGA or CGU or CGC or CGA or CGG) will produce a selectable pause state.  **Pausing Law**: Glycine GGG combined with Arginine (CGG or CGU or CGA or CGC) or Serine(UCG or UCA or UCU or UCC) will produce a selectable pause state.  **Pausing law:** Glycine GGA combined with Arginine (CGU or CGA or AGA or CGC) will produce a selectable pause state.  **Pausing Law**: Glycine GGA combined with any Serine will produce a pause state.  **Pausing Law:** Glycine GGC combined with Arginine AGG will produce a selectable stop state.  **Pausing Law**: Glycine GGU combined with any Glu or Val or Asp will produce a stop state.  **Pausing law:** Glycine GGG combined with Arginine(AGG or AGA) will produce a selectable stop state.  **Pausing law**: Glycine GGG combined with Serine(AGU or AGC) will produce a selectable stop state.  **Pausing Law:** Glycine GGA combined with Arginine(CGG or AGG) will produce a selectable stop state.  **Pausing Law**: Glycine GGU combined with Arginine AGG will produce a selectable stop state. |
| --- |

**Supplementary Table 20** Logic Laws for Glycine as primary codon
